# Elderly Perception of Distance to the Grocery Store as a Reason for Feeling Food Insecurity—Can Food Policy Limit This?

**DOI:** 10.3390/nu12103191

**Published:** 2020-10-19

**Authors:** Robert Gajda, Marzena Jeżewska-Zychowicz

**Affiliations:** 1 Department of Human Nutrition, Faculty of Biotechnology and Food Sciences, Wroclaw University of Life Sciences, Norwida 25, 50-375 Wroclaw, Poland; 2 Department of Food Market and Consumer Research, Institute of Human Nutrition Sciences, Warsaw University of Life Sciences (SGGW-WULS), Nowoursynowska 159C, 02-776 Warsaw, Poland; marzena_jezewska_zychowicz@sggw.edu.pl

**Keywords:** food insecurity, elderly people, food policy, health policy, socio-demographic features

## Abstract

The sense of food insecurity in a group of elderly people may be determined by the perception of distance to food outlets. The aim of the study was to assess the relationship between the perception of food insecurity by the elderly and their perception of the distance between the places of residence and food purchase. A cross-sectional quantitative survey was carried out in 2019–2020 amongst 762 Polish elderly living in Świętokrzyskie and Śląskie Voivodeship. The assessment of the relationships between the perceived food insecurity due to living too far away from the grocery shops and socio-demographic features was performed using multiple correspondence analysis (MCA), chi-square tests, and Phi and Cramér’s V coefficients. Too great a distance to the place of food purchase was the cause of a lack of a sense of food security in 20.5% of the study sample, especially in men, people aged 75 and more, people living in a rural environment and people living alone. People reporting a lack of food due to the distance to the place of purchase showed socio-demographic characteristics similar to those of people declaring a lack of a sense of food security. Reduced food consumption due to the distance from the place of sale, including of fish (24.8%), some fruits (18.9%) and vegetables (15.4%) and beef (17.3%), may contribute to the deterioration of the diet and, as a result, health conditions. Including access to places of the sale of food in food policy as a factor contributing to ensuring the food security of older people can help to maintain a better quality of life and avoid exclusion. Especially in a situation of limited independence, food insecurity in elders due to causes other than financial limitations should be a focus of food policy.

## 1. Introduction

The aging of societies has increased in recent decades due to an increase in life expectancy and a decrease in the fertility rate [1]. According to the forecasts of the Central Statistical Office (GUS) in Poland, the share of older people (60 and more) in the population of Poland will increase from 25.9% in 2020 to 40.4% in 2050 [2]. Taking into account the economic and social costs involved with the aging of the population, the need to identify risk factors in order to protect against age-related health problems and disability seems to be obvious [3]. One such factor is food insecurity, understood as the limited or uncertain availability of nutritionally adequate and health-safe food or an uncertain ability to obtain acceptable food in a socially acceptable way [4]. In a large group of elderly households, there is a risk of food insecurity [5,6,7,8,9]. Its consequence is many harmful health effects [10,11,12].

In a group of elderly people, food safety is determined by, among others, physical functioning and physical activity [13], socio-economic status [14,15], social relations, social capital and, a little, social support [16,17,18,19,20] but also the distance of shops or supermarkets from the place of residence [21,22,23]. The research conducted to date shows that the distance is one of the most important factors determining the choice of a retail outlet [24,25,26,27,28]. At the same time, the definition of food safety [29] refers to physical, economic and social accessibility, but none of these dimensions literally takes into account the consumer’s accessibility to food sales points, often referred to as “spatial” or “geographical” [30].

There is a growing interest in the environmental context of purchasing behaviour, which includes both social and physical issues [31]. According to Glanz et al. [32], there is a distinction between the social and individual contexts in purchasing behaviour. The first covers the number, type, location and availability of food outlets, while the individual context is determined by the perception of food and its surroundings, e.g., shops or eateries, in terms of the price, promotion, quality or nutritional value of food. The availability of food outlets can be described by reference to their density (number of food outlets in an administratively defined area) and proximity (the distance that is determined between a person’s place of residence and the nearest food sales point). The latter can be measured in a straight line (Euclidean distance) or by travel time [31]. Areas with poor access to food (low density or long distance), making it difficult to buy healthy and inexpensive food, are called “food deserts” [33]. Moreover, there is a growing interest in the purchasing behaviour of older people, mainly due to its health consequences and the deterioration in the quality of life of these people [10,11,12].

Previous research has shown that the elderly are less privileged in terms of food choices [34], their purchasing experiences [35,36] and planning the location of supermarkets [37,38,39]. Fitch [40] identifies older people and people with disabilities as consumer groups more likely to experience food-related purchasing problems. Older people are at a disadvantage regarding access not only to supermarkets but also to smaller food stores [25,41]. The distance between the place of purchase of food and the place of residence of the elderly determines their ability to purchase food and so eat it, thus providing adequate nutrients and health [21,22,23]. Food restrictions, both physical and economic, can also cause social inequalities [42], which is revealed, among others, in diet-related health outcomes [33,43]. Thus, in both the food and health policy of the state, the problem of accessing places to buy food in the group of elderly people, especially those living alone, should find its rightful place.

In Poland, social policy towards the elderly is implemented under the obligation resulting from the Act of 11 September 2015 regarding the elderly. The latest activities are included in the document “Social policy towards the elderly 2030. Security—Participation—Solidarity”, adopted by the Council of Ministers on 26 October 2018 [44]. This document, for the first time, designed activities aimed at the elderly, which have the potential to indirectly reduce the feeling of food insecurity, such as “adapting the place of residence to functional possibilities”, “ensuring optimal access to health, rehabilitation, care and nursing services tailored to the needs” and a “system of support for informal carers by public institutions”. Nevertheless, the recipients of these activities are only “dependent elderly people”, which significantly limits the possibilities for the use of this help by elderly people with various physical limitations who, however, are not dependent. Restrictions on access to food due to a long distance to shops may be partially reduced by the possibility of using various programmes targeted at the elderly, for example, “Care 75+”, the aim of which is to provide lonely elderly people aged 75 and more with support and assistance adequate for the needs resulting from their age and health condition as part of care services. In addition, there are programmes that directly offer food aid to low-income people, including the elderly, such as “State aid for nutrition”, “Operational Program Food Aid” and “Senior+”. The possibility of using funds under these programmes (increased economic availability), as well as the help of an informal guardian, enables the food security of the elderly to be increased. Food programmes are aimed at all people regardless of age, while the “Care 75+” programme focuses on medical services. In order to determine to what extent the presented actions are helpful in preventing the deterioration of the quality of life of older people, there is a need to assess the needs related to ensuring food security, both in the objective and subjective dimensions. A food policy aimed at the food security of older people should take into account objective indicators (density and distance to sales points) but also how the availability of food is perceived by these people. Research on the economic availability of food dominates [5,6,9,45,46], while the availability of food in subjective terms has so far rarely been the subject of research. In addition, some results show inconsistencies as to the relationship between access to stores and the level of food consumption and health in the elderly group [47,48,49] or lack thereof [50].

Ensuring food security in the elderly group should be an important element of the food policy in every country. One of the elements of this security is the availability of food selling points, which, due to the physical and economic limitations of older people, may be important to ensure. The aim of the study was, therefore, to assess the relationship between the perception of food insecurity by the elderly with different socio-demographic characteristics and their perception of the distance between the place of residence and food purchase in general. Moreover, changes in the way of satisfying the nutritional needs of elderly people were assessed, which resulted from limitations in the availability of food purchase places. The following research hypotheses were formulated: (1) limitations in the availability of food resulting from long distances of the residences of the elderly from the places of purchase contribute to increasing the feeling of food insecurity; (2) long distances between the places of residence of the elderly and the places of purchase reduce the availability of food, including basic food products; (3) both the sense of food insecurity and the experience of food shortage in the households of older people are determined by their socio-demographic characteristics.

## 2. Materials and Methods

### 2.1. Study Design and Sample

The survey was carried out at the turn of 2018 and 2019 in a group of people aged 65 and over. The study sample was selected using the snowball method. A total of 1150 questionnaires were distributed in 16 senior clubs in the Świętokrzyskie Voivodeship (the city of Kielce, Kielce and Sandomierz poviats) and in the Śląskie Voivodeship (the city of Częstochowa and Częstochowa poviat). The recruitment criterion was age; the respondents were 65 years of age and over. Those who agreed to participate in the study were asked to provide the questionnaire to people from their place of residence who met the age criterion. In all, 798 questionnaires were collected, of which 36 were eliminated due to missing answers. The study sample consisted of 762 people, including 445 from the Świętokrzyskie Voivodeship and 317 from the Śląskie Voivodeship. The characteristics of the study sample, taking into account socio-demographic features, are presented in Table 1.

The study was conducted in accordance with the Helsinki Declaration [51]. Informed consent to participate in the study was collected from all participants.

### 2.2. Questionnaire

The questionnaire included questions about:

1. Perceived worries related to the availability of food in the last month because of living at a great distance from shops and hypermarkets (Worries about food availability—W_F) (a question with no/yes answers: In the last month, have you been afraid that your household will run out of food because you live too far from shops and hypermarkets?).
2. The occurrence in the last month of the following consequences of living too far from the place for purchasing food, such as: (1) a lack of basic food products (e.g., bread, butter, milk or eggs) (Change in basic food availability—C_F) (a question with no/yes answers: In the last month, did you run out of basic food products (e.g., bread, butter, milk, eggs, etc.) in your household because you lived too far from shops and hypermarkets?) and (2) skipping any meal (Change—skipping meals—C_SM) (a question with no/yes answers: In the last month, did you have to skip a meal in your household because you live too far from shops and hypermarkets?).
3. The occurrence of a situation where there was a lack of food in the household, which resulted from living far away from the place of its purchase (What food products did you not have in your household on a daily basis because you live too far from shops and hypermarkets?). Twenty-seven groups of food products were included: bread; flour, groats or pasta; milk; fermented milk drinks, e.g., yoghurts and kefirs; cottage cheese (including homogenized and granulated cheese); yellow cheeses (including blue and cream cheeses); butter; margarine for spreads; lard, bacon or other animal fats; oil or olive oil; beef meat; pork meat; poultry meat; fish or fish preparations; fruits; fruit preserves, e.g., jam, and preserves, including honey; all vegetables; vegetable preserves, e.g., frozen, pickled and canned; legumes (dry), e.g., peas, beans, soybeans and lentils; sweets or cakes; sugar; mineral water; fruit, vegetable or vegetable and fruit juices; carbonated drinks, e.g., Coca-Cola, Fanta, Sprite and Orangeade; alcoholic drinks.

The questionnaire also included questions about socio-demographic characteristics, i.e., gender, age, place of residence, region of residence and the personal composition of the household.

### 2.3. Statistical Analysis

Descriptive statistics, including frequency distributions and cross-tabulations, were determined. The chi-square test was used to examine the differences between categorical variables.

**Figure 1:**
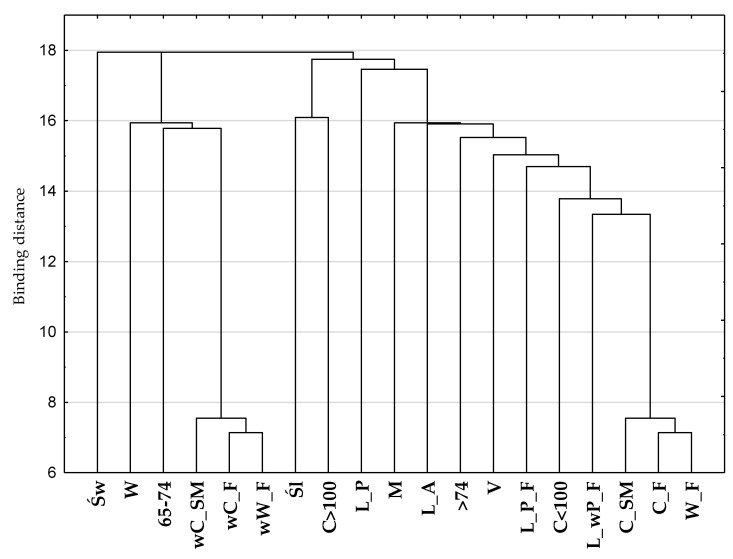
The hierarchical classification of variables describing food insecurity and selected socio-demographic features. W_F—worries about food availability; C_F—change in basic food availability; C_SM—change—skipping meals; wW_F—without worries about food availability; wC_F—without a change in basic food availability; wC_SM—without change—skipping meals; W—woman; M—man; 65–74—age in years; >74—aged 75 and over; V—village; C < 100—city with up to 100,000 inhabitants; C > 100—city with over 100,000 inhabitants; Św—Świętokrzyskie Voivodeship; Śl—Śląskie Voivodeship; L_A—I live alone; L_P—I live with a partner; L_wP_F—I live without a partner but with my family; L_P_F—I live with a partner and my family.

A multiple correspondence analysis (MCA) was used to identify the relationship between the categorical variables describing food insecurity (W_F; C_F; C_SM; wW_F—without worries about food availability; wC_F—without a change in basic food availability; wC_SM—without a change—skipping meals) and socio-demographic characteristics (gender, age, place of residence, region and household composition). In the analysis, the Burt matrix was used, and the cumulative percentage of inertia and the scree criterion were adopted as the criteria for selecting the number of dimensions of the projecting space of the variables [52]. The percentage of inertia for the first and second dimensions was 12.68 and 27.43, respectively. On the screen diagram, a clear collapse of the straight line occurred at the second point, which indicates the number of projection space dimensions. The own value for the two dimensions was 0.18. Based on both criteria, a two-dimensional projection was used for graphic presentation. In order to interpret the obtained results, a hierarchical classification of variables was made using the Ward’s method, which estimates the distance between sets (clusters) of variables using the analysis of variance [52]—Figure 1.

The relationships between variables were assessed using the Phi (Φ) coefficient when both variables were nominal, whereas Cramer’s V coefficient was used when one of them was ordinal [53].

Statistical analysis was performed using the statistical program STATISTICA 13.1 (version 13.1 PL; StatSoft Inc., Tulsa, OK, USA; StatSoft, Krakow, Poland). [54].

## 3. Results

**Figure 2:**
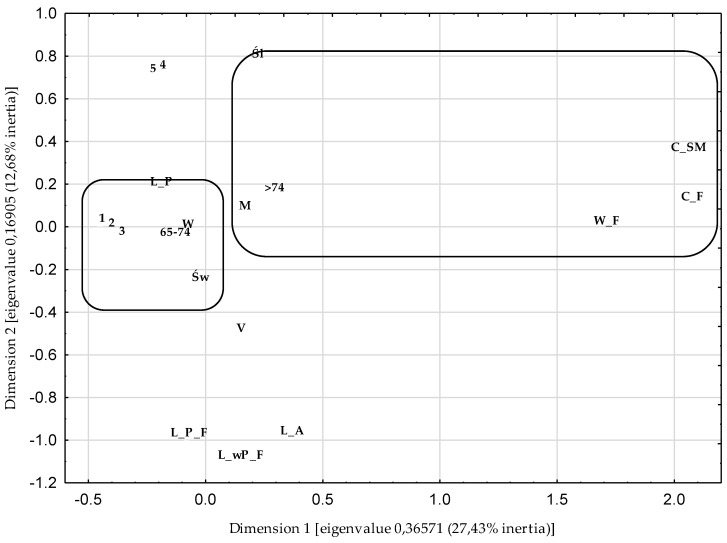
The structure of the relationship between the variables describing food insecurity and the socio-demographic features of the studied group (multiple correspondence analysis (MCA). W_F—worries about food availability; C_F—change in basic food availability; C_SM—change—skipping meals; 1—without worries about food availability (wW_F); 2—without a change in basic food availability (wC_F); 3—without change—skipping meals (wC_SM); W—woman; M—man; 65–74—age in years; >74—aged 75 and over; V—village; 4—city with up to 100,000 inhabitants (C < 100); 5—city with over 100,000 inhabitants (C > 100); Św—Świętokrzyskie Voivodeship; Śl—Śląskie Voivodeship; L_A—I live alone; L_P—I live with a partner; L_wP_F—I live without a partner but with my family; L_P_F—I live with a partner and my family.

The structures of the relationships between the variables describing the perception of food insecurity, taking into account long distances from the places of purchasing food to the places of residence, and socio-demographic features are presented in Figure 2. Using Ward’s hierarchical classification method, two collections were selected. One collection consisted of people who declared a lack of a sense of food security in their households (W_F, C_F, and C_SM) and at the same time were characterized by features such as: men, aged 75 and more, and living in the Śląskie Voivodeship. This collection includes all categories of the variable: place of residence and household structure. The second collection included those respondents who declared food security (wW_F, wC_F and wC_SM). They were women, people aged 65–74 and people living in the Świętokrzyskie Voivodeship.

Worries about food availability due to living too far from the place for purchasing food were declared by more respondents from the Śląskie Voivodeship (*p* < 0.05; Φ = 0.53), more men than women (*p* < 0.05; Φ = 0.36), more people aged 75 and over than those aged 65–74 (*p* < 0.05; Φ = 0.38), more people living in the countryside (*p* < 0.05; Cramer’s *V* = 0.23), and also the largest number of people living alone (*p* < 0.05; Cramer’s *V* = 0.11).

Changes in basic food availability due to living too far from the place for purchasing food were declared by twice as many respondents from the Śląskie Voivodeship (*p* < 0.05; Φ = 0.53), more men than women (*p* < 0.05; Φ = 0.38), more people aged 75 and over than those aged 65–74 (*p* < 0.05; Φ = 0.40), almost twice as many people living in the countryside as compared to the city (*p* < 0.05; Cramer’s *V* = 0.17), also the largest number of people living alone (*p* < 0.05; Cramer’s *V* = 0.13).

Similar differences were observed in the case of the declaration of changes regarding skipping meals. Such changes due to living far away from the place where food was purchased were declared by more people from the Śląskie Voivodeship (*p* < 0.05; Φ = 0.58), more men than women (*p* < 0.05; Φ = 0.37), more people aged 75 and over than those aged 65–74 (*p* < 0.05; Φ = 0.39), more people living in rural and urban areas with up to 100,000 inhabitants compared to a large city (*p* < 0.05; Cramer’s *V* = 0.17), and also more people living alone than others (*p* < 0.05; Cramer’s *V* = 0.13)—Table 2.

Worries about food availability due to living too far from the place for purchasing food were declared by more respondents from the Śląskie Voivodeship (*p* < 0.05; Φ = 0.53), more men than women (*p* < 0.05; Φ = 0.36), more people aged 75 and over than those aged 65–74 (*p* < 0.05; Φ = 0.38), more people living in the countryside (*p* < 0.05; Cramer’s *V* = 0.23), and also the largest number of people living alone (*p* < 0.05; Cramer’s *V* = 0.11).

The large distance between the place of residence and the place for the purchase of food was indicated by the highest percentage of elderly people as the reason for the lack of fish and fish products in their daily diet (24.8%), some fruit (18.9%), beef meat (17.3%) and some vegetables (15.4%). The products whose availability was to a small extent dependent on the distance to a place of purchase included fermented milk drinks, mineral water, fruit preserves, oil, olive oil, milk, bread, vegetables, spreadable margarine, sugar, flour and groats—Table 3.

In the case of the group of products, the lack of which was reported by the most people, different indications were shown, taking into account gender, age, place of residence and household composition (Table 3). The lack of these products was indicated by more men than women (some fruits, beef and some vegetables), more people over 74 (beef and some vegetables), rural residents (fish and their products, some fruit, beef and some vegetables), people living in the Śląskie Voivodeship (fish and their preserves, some fruit, beef and some vegetables) and people living without a partner with their family (fish and their preserves, some fruit, beef and some vegetables).

In the case of food products, the lack of which was recorded by a smaller percentage of the study sample, it was shown that more women than men reported the lack of such products in their households as milk, fermented milk drinks and cottage cheese; all fruits and vegetables or their preparations; legumes; animal fats and margarine for spreads; and flours, cereals, pasta, bread and sugar. Compared to those aged 65–74, fewer people aged 75 and more indicated animal fats, vegetable products and fermented milk drinks as unavailable due to large distances from places of sale. More people from the Świętokrzyskie Voivodeship indicated a lack of such products as animal fats, margarine for spreading and all fruit. On the other hand, a larger percentage of people living without a partner but with their family reported shortages of such food products as poultry meat, legumes, juices, milk, cheese, sugar, flour, groats and pasta. In turn, more people living alone experienced a shortage of such food products as bread, sweets and cakes; all vegetables and vegetable preserves; all fruit and fruit preserves; fermented milk drinks; and mineral water. In the case of people living with a partner and family, only a higher percentage of indications about the lack of margarine for spreading was shown. However, it has not been shown that more elderly people living with a partner, compared to other people, experienced food shortages on a daily basis (Table 3).

In the study sample, 16% of people declared a lack of availability of alcoholic beverages due to large distances from places of sale. This group included more men (17.5%), people over 74 (19.1%), rural residents (23.3%), people from the Śląskie Voivodeship (16.6%), and people living without a partner but with their family (19.1%)—Table 3.

## 4. Discussion

Food insecurity within the elderly can be the result of a number of reasons, including a lack of money for food, health or mobility limitations, and also not enough food due to transportation limitations [55], making the distance from the home to the store more important. Although elderly food insecurity can be considered in four dimensions—quantitative, qualitative, psychological and social—only the quantitative and psychological ones were considered in this study. The latter relates to the currently available food for consumption. Reduced food stocks and eating less food than usual may result in hunger. The psychological component of food insecurity refers to the elders’ knowledge and perception of their food situation and to their feelings about it. Knowing and perceiving the uncertainty of the food situation and the lack of adequate foods for health leads to feelings of worry and anxiety. Finally, the presence of foodstores nearby and public transport services is important from the perspective of food insecurity and thus independent aging [56]. Our study found that restrictions in the availability of food resulting from the distance between the place of residence of the elderly and the place of purchase were more often associated with declaring concerns related to the availability of food (psychological component of food insecurity), while slightly fewer people reported specific changes related to the availability of food in their households (quantitative component of food insecurity). In the study sample, approximately 15% of people experienced changes in consumption due to a lack of food because of the distance to the store.

The reasons for the difficulties related to the long distances of the residences of the elderly from the places for purchasing food are limited mobility and transportation, but also the locations of supermarkets in the suburbs [57,58,59]. This may hinder access to these places, thus limit the availability of the usually lower-priced food sold there, and consequently contribute to food shortages [58]. In this study, a large distance between the place of residence and a place where food was sold resulted in a lack of products such as fish and fish products, some fruits, beef meat and some vegetables in the daily diet. Limiting the consumption of these products may lead to a reduction in the nutritional value of the food rations of the elderly; promote deficiencies in vitamins, minerals, dietary fibre and unsaturated fatty acids [60,61]; and, as a result, maybe increase the risk of, for example, cardiovascular diseases, obesity, hypertension, type 2 diabetes and cancer, and exacerbate the symptoms of existing diseases [62,63,64,65]. Research by Morland et al. [66] suggests that living in areas with easy access to food sales points improves the quality of the diet, especially in terms of the structure of the consumption of vegetables, fruits, total fats and saturated fatty acids. This is especially noticeable in the case of easy access to supermarkets offering food at lower prices.

The results of the Public Opinion Research Centre (CBOS) study [67] confirm the obtained results on food insecurity in the context of the possibility of purchasing food among Polish elderly. Difficulties in carrying out everyday activities were declared by 22% of people aged 75 and above, including mainly people with low education, and people dissatisfied with the material conditions of their households, but also people living alone. In our study, people who required assistance in activities related to the purchase of food due to the large distance from shops were, in particular, men, people aged 75 and above, rural residents and people living alone or with their family but without a partner. Nevertheless, the CBOS study also shows that 94% of older people who cannot cope with everyday activities can count on support from their immediate or distant family, while older people use institutional forms of support less often. The majority of elderly people with difficulties in carrying out daily activities received support in running a household (80%), which may explain the higher percentage of people with concerns about food insecurity in our study who reported food restrictions. The reason for this difference may be the lack of funds for the purchase of food or the use of transport, which is confirmed by the fact that more than half of the respondents (55%) declaring that they needed financial support did not receive such assistance [67].

The failure of the elderly to use assistance in coping with everyday activities, including food shopping, may result from insufficient information on the availability of such assistance. The evaluation of the activities of social welfare centres shows that information on the possibility of obtaining help was published on the websites of the centres or city offices, in the Public Information Bulletin or on noticeboards at the premises of these units. This method of informing clients, who are usually elderly and often do not use modern communication techniques, and are often physically disabled, may not be effective [68]. In such a situation, actions taken under the food policy should focus not only on specific help provided to people in need of it, but also on effective information about the possibility of using help.

The assessment of food insecurity made by the elderly, taking into account the distance from home to the place of sale, shows a large diversity in terms of socio-demographic characteristics. This applies to both perceived concerns related to the availability of food and declarations of specific changes in the availability of basic food and the omission of certain meals. The people most affected by food security threats are the inhabitants of the Śląskie Voivodeship, people from rural areas, men, people living alone, and those aged 75 and above. Comparable results concerning socio-demographic conditions in ensuring food security were obtained in other studies [6,45,46,69].

The explanation for the differences in the perception of food insecurity between the inhabitants of the Śląskie and Świętokrzyskie Voivodeships requires a very detailed analysis of the situation in these regions and, above all, the level of household income, the location of shops and the communication system, but also the level of social assistance, the organization of which is the responsibility of local authorities. The differences between the two voivodeships are noted in the published statistical data [70,71,72]. The research was local in nature, but the differences between the two spatially adjacent regions indicate a significant importance of the regional specificity in the perception of food insecurity by the elderly. The specificity and regional differences of the Świętokrzyskie and Śląskie Voivodeships in terms of conditions and quality of life have been demonstrated in many studies [73,74,75]. However, no Polish research has shown regional differentiation as the reason for food insecurity in households of older people. Hence, a developed diagnosis of the regional situation has a chance to increase the effectiveness of actions undertaken in the implementation of national food policy recommendations.

The characteristics of people experiencing food insecurity, as shown in our study, are similar to those in the situation in other countries. The studies conducted to date clearly indicate that life in rural areas is a risk factor threatening food security [45,46,69]. In the rural environment, more concerns about the availability of food were declared, and more changes in households were revealed as being due to great distances to places where food was sold. According to Quandt et al. [76], for older adults living in rural areas, several factors reduce access to healthy food in relation to the general population of older adults, including lower incomes and poorer health than their urban and suburban counterparts, higher costs and the limited selection of food. However, elderly living in a rural environment may be protected against food insecurity by the practice of gardening and other forms of home food production. Those of older age and living in the countryside have limited access to shops due to limitations in their own or public transport (e.g., a lack of own car or the ability to drive it) [77]. Restrictions in movement resulting from diseases of the locomotor system and, at the same time, poor public transport in rural areas [77] increase dependence on the help of third parties [20,47,78,79]. Moreover, living alone, especially in a rural setting, can exacerbate this limitation [80,81], which is confirmed by the obtained results.

Men reported greater problems with satisfying food security resulting from the availability of food purchase places. This is confirmed by the results of other studies [47]; however, there are also studies that have shown a greater exposure to food insecurity among women [82]. A greater threat to meeting food security was observed among people aged 75 and over [83,84,85].

Additionally, the lower availability of food products due to large distance from shops was conditioned by socio-demographic characteristics. As in the case of feeling anxious about the availability of food, men, people over 74, inhabitants of rural areas, people living in the Śląskie Voivodeship and people living without a partner with their family were the groups most vulnerable to the shortage of certain products due to large distances from their places of residence to places of food purchase.

Physical access to stores is an important reason for older consumers to choose where to buy [28]. A long distance from the place of residence to a place for purchasing food and a lack of transport increase the dependence of the elderly on other people, limit the choice of food and, at the same time, encourage the use of free buses from commercial networks [27]. Thus, the presence of shops nearby and public transport services, especially for people who do not have a driving licence or no longer have one, is necessary for independent aging [56] and also to meet the need for food security. Older car-less people living in an area with numerous amenities and services and good public transport available may perceive a good quality of life, despite the more limited space for activities [86]. However, proximity to the place of living, frequently cited in the literature as a key attribute influencing store choice [87], was not the most important factor in choosing a store. The most decisive factor for store choice was the price level of the foods purchased, followed by habits and routines from the past, and finally, 24% of respondents indicated the proximity to the place of living, which may suggest that older consumers may be adapting to their difficulties. Thus, in efforts to improve the food security of the elderly, the distance from the place of residence to the place where food is purchased is one of the key elements, especially in relation to the oldest people living alone and in a rural environment. However, it is known that the demand in the food desert areas is too small to encourage businesses to open new stores in places where the elderly do not have physical access to food. Therefore, to increase the sense of their food security, it would be necessary to develop other channels of distribution in food-insecure areas. Online grocery sales may not be suitable for older people due to their limited use of modern technologies and little confidence in them [68,88]. Thus, government-supported food distribution programs seem to be a good solution, especially since they have already been proven useful in various situations of food shortage, primarily those resulting from financial problems. However, it is still necessary to develop new effective solutions, whose introduction should be preceded by research on their acceptance amongst older people. Further research is also needed to help bridge the gap between academics, policymakers and practitioners in the area of food security [89].

One of the limitations of our study is that it only takes into account the occurrence of food insecurity in households of the elderly but does not address its intensity. Moreover, the perceptions of food insecurity and proximity to food stores were not considered in terms of the size of financial resources and/or the level of household poverty. The latter could have influenced the perception of distance, e.g., the inability to use one’s own means of transport. The distance from the place of food purchase was not determined, but only a subjective assessment of this distance in general was made. Moreover, the study was cross-sectional, and the cause-and-effect relationship between food insecurity and distance perception cannot be fully established. Due to the lack of representativeness of the study group (only two regions), the results of the study cannot be applied to the entire Polish population.

## 5. Conclusions

Too large a distance from the place of residence to the place where food was purchased was the cause of a lack of a sense of food security in the studied group of elderly people, especially in the groups of men, people aged 75 and more, those living in a rural environment and those living alone. People informing about a lack of food due to living too far from the place of purchase had socio-demographic characteristics similar to people declaring a lack of a sense of food security. Restricted food consumption due to distance to the place of sale, including of fish, some fruit and vegetables, and beef, may contribute to deterioration of the diet and, as a result, the health of older people.

Including distance to food sales points in food policy as a factor contributing to the food security of older people can help to ensure a better quality of life. The self-purchase of food helps to meet physiological needs but also to maintain contact with the community and avoid exclusion. In a situation of limited independence, food insecurity in elders due to causes other than financial limitations should be a focus of food policy.

## Figures and Tables

**Table 1:** Characteristics of the studied group, taking into account selected socio-demographic characteristics (%).

Socio-Demographic Characteristics	Total	
	*N* = 762	(%)
Gender		
Woman	528	69.3
Man	234	30.7
Age		
65–74 years	532	69.8
75 and more years	230	30.2
Place of residence		
Village	245	32.2
City < 100,000 inhabitants	123	16.1
City ≥ 100,000 inhabitants	394	51.7
Region of residence		
Świętokrzyskie Voivodeship	445	58.4
Śląskie Voivodeship	317	41.6
Household composition		
I live alone	237	31.1
I live with my partner	300	39.4
I live without a partner but with my family	89	11.7
I live with a partner and my family	136	17.8

**Table 2:** Opinions on the food insecurity of the household, taking into account selected socio-demographic characteristics of the studied group (%).

Socio-Demographic Features	Opinions on Food Insecurity					
	W_F * (*n* = 156)		C_F * (*n* = 123)		C_SM * (*n* = 112)	
	%	Φ/Cramer’s *V*	%	Φ/Cramer’s *V*	%	Φ/Cramer’s *V*
Total	20.5		16.1		14.7	
Gender		0.36 **		0.38 **		0.37 **
Men	18.9 ^a^		14.2 ^a^		12.5 ^a^	
Women	23.9 ^b^		20.5 ^b^		19.7 ^b^	
Age		0.38 **		0.40 **		0.39 **
65–74 years	17.1		13.3 ^a^		13.2 ^a^	
75 and more years	28.3		22.6 ^b^		18.3 ^b^	
Place of residence		0.23 ***		0.17 ***		0.10 ***
Village	33.9 ^a^		24.9 ^a^		19.0 ^a^	
City < 100,000 inhabitants	14.6 ^a^		15.4 ^a^		17.9 ^a^	
Miasto ≥ 100,000 inhabitants	13.6 ^b^		10.9 ^b^		11.2 ^b^	
Region of residence		0.53 **		0.53 **		0.58 **
Świętokrzyskie Voivodeship	14.2 ^a^		10.4 ^a^		12.6 ^a^	
Śląskie Voivodeship	24.9 ^b^		20.2 ^b^		16.2 ^b^	
Household composition		0.11 ***		0.13 ***		0.11 ***
I live alone	26.2 ^a^		22.4 ^a^		20.2 ^a^	
I live with my partner	16.0 ^b^		12.0 ^b^		12.3 ^b^	
I live without a partner but with my family	22.5 ^c^		19.1 ^c^		12.4 ^b^	
I live with a partner and my family	19.1 ^d^		12.5 ^b^		11.8 ^b^	

^a–d^ Statistically significant differences between particular categories of socio-demographic features are marked with different letters (chi-squared test, *p* < 0.05). * W_F—worries about food availability; C_F—change in basic food availability; C_SM—change—skipping meals; ** Phi coefficient (Φ); *** Cramer’s V coefficient.

**Table 3:** Declared shortages of food products due to long distances of living from the places for their purchase, taking into account selected socio-demographic features (%).

Product Categories	Total	Socio-Demographic Features												
		Gender		Age (Years)		Place of Residence			Region of Residence		Household Composition			
		W * (N = 528)	M (N = 234)	65–74 (N = 532)	>74 (N = 230)	V (N = 245)	C < 100 (N = 123)	C > 100 (N = 394)	Św (N = 445)	Śl (*N* = 319)	L_A (*N* = 237)	L_P (*N* = 300)	L_wP_F (*N* = 89)	L_P_F (*N* = 136)
Fish and their preserves	24.8	24.8	24.8	22.0	31.3	37.5 ^a^	22.0 ^a^	17.8 ^b^	19.6 ^a^	28.5 ^b^	28.3 ^a^	19.7 ^b^	37.0 ^a^	22.0 ^c^
Some fruits	18.9	17.1 ^a^	19.7 ^b^	17.1	23.0	31.4 ^a^	14.6 ^a^	12.4 ^b^	15.1 ^a^	21.6 ^b^	19.8 ^a^	13.7 ^a^	29.2 ^b^	22.1 ^c^
Beef meat	17.3	17.2 ^a^	17.5 ^b^	15.0 ^a^	22.6 ^b^	35.1 ^a^	14.6 ^a^	7.1 ^b^	12.9 ^a^	20.4 ^b^	15.2 ^a^	13.7 ^a^	29.2 ^b^	21.3 ^c^
Alcoholic drinks	16.0	15.3 ^a^	17.5 ^b^	14.7 ^a^	19.1 ^b^	23.3 ^a^	17.9 ^a^	10.9 ^b^	15.1 ^a^	16.6 ^b^	13.5 ^a^	17.7 ^a^	19.1 ^b^	14.7 ^a^
Some vegetables	15.4	15.2 ^a^	15.8 ^b^	15.0 ^a^	16.1 ^b^	24.9 ^a^	11.4 ^a^	10.7 ^b^	10.4 ^a^	18.9 ^b^	12.7 ^a^	14.3 ^a^	20.2 ^b^	19.1 ^b^
Carbonated drinks	10.1	9.7 ^a^	11.1 ^b^	8.5 ^a^	13.9 ^b^	16.3 ^a^	7.3 ^b^	5.8 ^c^	8.5 ^a^	11.2 ^b^	8.9 ^a^	10.0 ^a^	12.4 ^a^	11.0 ^a^
Sweets and cakes	7.9	7.4 ^a^	9.0 ^b^	6.6 ^a^	10.9 ^b^	11.8 ^a^	6.5 ^b^	5.8 ^c^	5.6 ^a^	9.4 ^b^	9.7 ^a^	6.3 ^b^	7.8 ^c^	8.1 ^c^
Pork meat	6.3	6.1 ^a^	6.8 ^b^	5.3 ^a^	8.7 ^b^	11.8 ^a^	4.1 ^b^	3.6 ^c^	3.5 ^a^	8.3 ^b^	7.2 ^a^	3.0 ^b^	10.1 ^c^	9.5 ^c^
Juices	5.8	5.7 ^a^	6.0 ^b^	4.7 ^a^	8.3 ^b^	10.2 ^a^	2.4 ^b^	4.1 ^c^	5.0 ^a^	6.3 ^b^	5.5 ^a^	3.7 ^a^	11.2 ^b^	7.4 ^c^
Yellow cheeses	5.4	4.7 ^a^	6.8 ^b^	4.7 ^a^	7.0 ^b^	12.7 ^a^	4.1 ^b^	1.3 ^c^	2.5 ^a^	7.4 ^b^	5.1 ^a^	3.7 ^a^	9.0 ^b^	7.4 ^c^
Legumes	5.4	5.8 ^a^	4.7 ^b^	4.7 ^a^	7.0 ^b^	12.7 ^a^	4.1 ^b^	1.3 ^c^	5.4	5.4	4.2 ^a^	4.0 ^a^	8.9 ^b^	8.0 ^c^
Cottage cheeses	4.9	4.9 ^a^	4.7 ^b^	3.4 ^a^	8.3 ^b^	8.9 ^a^	3.3 ^b^	2.8 ^c^	3.2 ^a^	6.1 ^b^	5.9 ^a^	3.7 ^b^	4.5 ^b^	5.9 ^a^
Animal fats	3.9	4.7 ^a^	2.1 ^b^	4.7 ^a^	2.2 ^b^	6.5 ^a^	1.6 ^b^	3.0 ^c^	5.4 ^a^	0.7 ^b^	2.1 ^a^	5.3 ^b^	2.2 ^a^	5.1 ^b^
Poultry meat	3.9	2.8 ^a^	6.4 ^b^	2.8 ^a^	6.5 ^b^	6.5 ^a^	2.4 ^b^	2.8 ^c^	2.5 ^a^	4.9 ^b^	4.2 ^a^	2.7 ^b^	7.9 ^c^	3.7 ^a^
Vegetable preserves	3.9	4.2 ^a^	3.4 ^b^	4.1 ^a^	3.5 ^b^	4.5 ^a^	5.7 ^b^	3.0 ^c^	3.2 ^a^	4.5 ^b^	4.6 ^a^	3.7 ^b^	3.4 ^b^	3.7 ^b^
Butter	3.8	3.2 ^a^	5.1 ^b^	3.4 ^a^	4.8 ^b^	6.9 ^a^	2.4 ^b^	2.3 ^c^	3.2 ^a^	4.3 ^b^	4.6 ^a^	3.0 ^b^	4.5 ^a^	3.7 ^c^
Fermented milk drinks	3.1	3.4 ^a^	2.6 ^b^	3.2 ^a^	3.0 ^b^	5.7 ^a^	4.1 ^b^	1.3 ^c^	1.3 ^a^	4.5 ^b^	3.8 ^a^	3.7 ^b^	3.4 ^b^	0.7 ^c^
Mineral water	2.9	2.7 ^a^	3.4 ^b^	1.7 ^a^	5.7 ^b^	4.1 ^a^	2.4 ^b^	2.3 ^c^	2.5 ^a^	3.1 ^b^	5.1 ^a^	1.0 ^b^	4.5 ^c^	2.2^d^
Fruit preserves	2.9	3.2 ^a^	2.1 ^b^	2.4 ^a^	3.9 ^b^	3.7 ^a^	2.4 ^b^	2.5 ^c^	2.2 ^a^	3.4 ^b^	3.8 ^a^	2.7 ^b^	1.1 ^c^	2.9
All fruits	2.8	3.0 ^a^	2.1 ^b^	2.3 ^a^	3.9 ^b^	4.1 ^a^	4.1 ^a^	1.5 ^c^	2.8 ^a^	2.7 ^b^	4.2 ^a^	2.0 ^b^	2.2 ^b^	2.2 ^b^
Oil or olive oil	2.6	2.7	2.7	1.9 ^a^	4.3 ^b^	3.7 ^a^	2.4 ^b^	2.0 ^c^	1.3 ^a^	3.6 ^b^	3.0 ^a^	2.3 ^b^	2.2 ^b^	2.9 ^a^
Milk	2.5	2.7 ^a^	2.1 ^b^	0.2 ^a^	4.8 ^b^	4.9 ^a^	0.8 ^b^	1.5 ^c^	1.3 ^a^	3.4 ^b^	3.4 ^a^	1.3 ^b^	6.7 ^c^	0.7^d^
Bread	2.2	2.3 ^a^	2.1 ^b^	1.1 ^a^	4.8 ^b^	2.4 ^a^	3.2 ^b^	1.8 ^c^	0.9 ^a^	3.1 ^b^	4.6 ^a^	0.7 ^b^	2.2 ^c^	1.5 ^c^
All vegetables	1.8	1.5 ^a^	0.4 ^b^	0.9 ^a^	1.7 ^b^	1.2 ^a^	2.4 ^b^	0.8 ^c^	0.9 ^a^	1.3 ^b^	2.5 ^a^	0.3 ^b^	0.0 ^b^	1.5 ^c^
Margarine for spreads	1.8	2.3 ^a^	0.9 ^b^	2.1 ^a^	1.3 ^b^	2.9 ^a^	1.6 ^b^	1.3 ^c^	2.5 ^a^	1.3 ^b^	1.7 ^a^	2.7 ^b^	0.0 ^c^	1.5 ^c^
Sugar	1.7	2.1 ^a^	0.9 ^b^	1.3 ^a^	2.6 ^b^	2.9 ^a^	0.0 ^b^	1.5 ^c^	1.3 ^a^	2.0 ^b^	2.1 ^a^	1.3 ^b^	3.4 ^c^	0.7^d^
Flour, groats, pasta	1.2	1.5 ^a^	0.4 ^b^	0.4 ^a^	3.0 ^b^	2.0 ^a^	0.0 ^b^	1.0 ^c^	0.6 ^a^	1.6 ^b^	1.3 ^a^	0.7 ^b^	2.4 ^c^	1.5 ^a^
Oil or olive oil	2.6	2.7	2.7	1.9 ^a^	4.3 ^b^	3.7 ^a^	2.4 ^b^	2.0 ^c^	1.3 ^a^	3.6 ^b^	3.0 ^a^	2.3 ^b^	2.2 ^b^	2.9 ^a^

^a–d^ Statistically significant differences between particular categories of socio-demographic features are marked with different letters (chi-square *p* < 0.05). * W—woman; M—man; 65–74—age in years; >74—aged 75 and over; V—village; C < 100—city with up to 100,000 inhabitants; C > 100—city with over 100,000 inhabitants. Św—Świętokrzyskie Voivodeship; Śl—Śląskie Voivodeship; L_A—I live alone; L_P—I live with a partner; L_wP_F—I live without a partner but with my family; L_P_F—I live with a partner and my family.

## References

[1] United Nations Population Division, Department of Economic and Social Affairs. http://www.un.org/en/development/desa/population/.

[2] Ludność w Wieku 60+. Struktura Demograficzna i Zdrowie. https://stat.gov.pl/obszary-tematyczne/ludnosc/ludnosc/ludnosc-w-wieku-60-struktura-demograficzna-i-zdrowie,24,1.html.

[3] Wang K., Bishop N.J. (2009). Social support and monetary resources as protective factors against food insecurity among older Americans: Findings from a health and retivement study. Food Secur..

[4] Anderson S.A. (1990). Core indicators of nutritional state for difficult-to-sample populations. J. Nutr..

[5] Gregório M.J., Rodrigues A.M., Graça P., de Sousa R.D., Dias S.S., Branco J.C., Ganhão H. (2018). Food insecurity is associated with low adherence to the Mediterranean diet and adverse health contitions in Portuguese adults. Front. Public Health.

[6] Leroux J., Morrison K., Rosenberg M. (2018). Prevalence and predictors of food insecurity among older people in Canada. Int. J. Environ. Res. Public Health.

[7] Nord M., Coleman-Jensen A., Andrews M., Carleson S. (2010). Household in the United States, 2009.

[8] Russell J., Flood V., Yeatman H., Mitchell P. (2014). Prevalence and risk factors of food insecurity among a kohort a folder Australians. J. Nutr. Health Aging.

[9] Tarasuk V., Fafard St-Germain A.A., Mitchell A. (2019). Geographic and socio-demographic predictors of household food insecurity in Canada, 2011–2012. BMC Public Health.

[10] Bishop N.J., Wang K. (2018). Food insecurity, comorbidity, and mobility limitations among older U.S. adults: Findings from the health and retirement study and health care and nutrition study. Prev. Med..

[11] Seligman H.K., Laraia B.A., Kushel M.B. (2010). Food insecurityis associated with chronic disease among low-income NHANES participants. J. Nutr..

[12] Ziliak J.P., Gundersen C. The Health Consequences of Senior Hunger in the United States: Evidence from the 1999–2010 NHANES. http://www.aaa1b.org/wp-content/uploads/2016/08/Health-Consequences-of-Food-Insecurity-final.pdf.

[13] Thompson J.L., Bentley G., Davis M., Coulson J., Stathi A., Fox K.R. (2011). Food shopping habits, physical activity and health-related indicators among adults aged >/=70 years. Public Health Nutr..

[14] Conklin A.I., Maguire E.R., Monsivais P. (2013). Economic determinants of diet in older adults: Systematic review. J. Epidemiol. Community Health.

[15] Dean W.R., Sharkey J.R. (2011). Food insecurity, social capital and perceived personal disparity in a predominantly rural region of Texas: An individual-level analysis. Soc. Sci. Med..

[16] Davis B.L., Grutzmacher S.K., Munger A.L. Utilization of social support among food insecure individuals: A qualitative exam-ination of network strategies and appraisals. J. Hunger Environ. Nutr..

[17] Garasky S., Morton L.W., Greder K.A. (2006). The effects of thelocal food environment and social support on rural food insecurity. J. Hunger Environ. Nutr..

[18] Sharifi N., Dolatian M., Mahmoodi Z., Abadi F.M.N., Mehrabi Y. (2017). The relationship between social support and food insecurity in pregnant women: A cross-sectional study. J. Clin. Diagn. Res..

[19] Vozoris N.T., Tarasuk V.S. (2003). Household food insufficiency is associated with poorer health. J. Nutr..

[20] Ramage-Morin P.L., Garriguet D. (2013). Nutritional risk among older Canadians. Health Rep..

[21] Caspi C.E., Sorensen G., Subramanian S.V., Kawachi I. (2012). The local food environment and diet: A systematic review. Health Place.

[22] Ishikawa M., Yokoyama T., Murayama N. (2013). Relationship between geographic factor induced food availability and foodintake status: A systematic review. Jpn. J. Nutr. Diet.

[23] Larson N.I., Story M.T., Nelson M.C. (2009). Neighborhood environments: Disparities in access to healthy foods in the U.S.. Am. J. Prev. Med..

[24] Grzybowska-Brzezińska M., Szmyt M. (2011). Wybrane obszary zachowań rynkowych seniorów. Zeszyty Naukowe Uniwersytetu Szczecińskiego. Ekon. Probl. Usług.

[25] Hare C. (2003). The food-shopping experience: A satisfaction survey of olderScottish consumers. J. Retail Distrib. Manag..

[26] Kowalczuk I. (2007). Zachowania nabywcze na rynku żywności osób w średnim i starszym wieku. ACTA Sci. Pol..

[27] Meneely L., Strugnell C., Burns A. (2009). Elderly consumer and their food store experiences. J. Retail. Consum. Serv..

[28] Whelan A., Wrigley N., Warm D., Cannings E. (2002). Life in a ‘food desert’. Urban Stud..

[29] (2009). The State of Food Insecurity in the World 2009. Economic Crises—Impacts and Lesson Learned.

[30] Sharkey J.R., Horel S. (2008). Neighborhood socioeconomic deprivation and minority composition are associated with better potential spatial access to the ground—Truthed food environment in a large rurel area. J. Nutr..

[31] Charreire H., Casey R., Salze P., Simion C., Chaix B., Banos A., Badariotti D., Weber C., Oppert J.-H. (2010). Measuring the food environment using geographical information system: A methodological rewiew. Public Health Nutr..

[32] Glanz K., Sallis J.F., Saelens B.E., Frank L.D. (2005). Healthy nutrition environments: Concepts and measures. Am. J. Health Promot..

[33] White M. (2007). Food access and obesity. Obes. Rev..

[34] Leighton C., Seaman C. (1997). The elderly food consumer: Disadvantaged?. J. Consum. Stud. Home Econ..

[35] Guy C. (2004). Neighbourhood retailing and food poverty: A case study in Cardiff. Int. J. Retail Distrib. Manag..

[36] Woodliffe L. (2004). Rethinking consumer disadvantage: The importance ofqualitative research. Int. J. Retail Distrib. Manag..

[37] Lang T., Caraher M. (1998). Access to healthy foods: Part II. Food poverty andshopping deserts: What are the implications for health promotion policy andpractice?. Health Educ. J..

[38] Szmigin I., Maddock S., Carrigan M. (2003). Conceptualising communityc.onsumption Farmers’ markets and the older consumer. Br. Food J..

[39] Kirkup M., De Kervenoael R., Hallsworth A., Clarke I., Jackson P., Perez-del-Aguila R. (2004). Inequalities in retail choice: Exploring consumer experiencesin suburban neighbourhoods. Int. J. Retail Distrib. Monag..

[40] Fitch D. (2004). Measuring convenience: Scots’ perception of local food and retailprovision. Int. J. Retail Distrib. Monag..

[41] Cummins S., Macintyre S. (1999). The location of food stores in urban areas: A casestudy in Glasgow. Br. Food J..

[42] Apparicio P., Abdelmajid M., Riva M., Shearmur R. (2008). Camparing alternative approaches to measuring the geographical accessibility of urban health services: Distance types and aggregation-error issues. Int. J. Health Geogr..

[43] Ford P.B., Dzewaltowski D.A. (2008). Disparities in obesity prevalence due to variation in the retail food environment: Three testable hypotheses. Nutr. Rev..

[44] Uchwała nr 161 Rady Ministrów z Dnia 26 Października 2018 r. w Sprawie Przyjęcia Dokumentu Polityka Społeczna Wobec Osób Starszych 2030. Bezpieczeństwo—Uczestnictwo—Solidarność. http://isap.sejm.gov.pl/isap.nsf/DocDetails.xsp?id=WMP20180001169.

[45] Haro-Mota R., Marceleño-Flores S., Bojórquez-Serrano J.I., Nájera-González O. (2016). La inseguridad alimentaria en El estado de Nayarit, México, y su asociación con factores socioeconómicos. Salud Publica Mex..

[46] Mundo-Rosas V., Méndez-Gómez Humarán I., Shamah-Lexy T. (2014). Caracterización de los hogares mexicanos en inseguridad alimentaria. Salud Publica Mex..

[47] Fukuda Y., Ishikawa M., Yokoyama T., Hayashi T., Nakaya T., Takemi Y., Kusama K., Yoshiike N., Nozue M., Yoshiba K. (2017). Physical and social determinants of dietary variety among older adults living alone in Japan. Geriatr. Gerontol. Int..

[48] Hanibuchi T., Kondo K., Nakaya T., Nakade M., Ojima T., Hirai H., Kawachi I. (2011). Neighborhood food environment and body mass index among Japanese older adults: Results from the Aichi Gerontological Evaluation Study (AGES). Int. J. Health Geogr..

[49] Murakami K., Sasaki S., Takahashi Y., Uenishi K. (2010). Japan Dietetic Students’ Study for Nutrition and Biomarkers Group. No meaningful association of neighborhood food store availability with dietary intake, body mass index, or waist circumference in young Japanese women. Nutr. Res..

[50] Yakushiji T., Asakawa T., Iwama N., Takahashi K., Tanaka K. (2015). Difficulties in Accessing Grocery Stores in a Super-Aged Society.

[51] (2013). World Medical Association Declaration of Helsinki: Ethical principles for medical research involving human subjects. JAMA J. Am. Med. Assoc..

[52] Stanisz A. (2006). Przystępny Kurs Statystyki. Analizy Wielowymiarowe.

[53] Stanisz A. (2006). Przystępny kurs Statystyki. Statystyki Podstawowe.

[54] StatSoft Poland Statistica 13.1. https://www.statsoft.pl/statistica_13/.

[55] Wendy S., Wolfe E.A., Frongillo P.V. (2003). Understanding the Experience of Food Insecurity by Elders Suggests Ways to Improve Its Measurement. J. Nutr..

[56] Rosenberg M., Everitt J. (2001). Planning for aging populations: Inside or outside the walls. Prog. Plann..

[57] Lee J.S., Frongillo E.A. (2001). Factors associated with food insecurity among US elderly persons: Importance of functional impairments. J. Gerontol. Ser. B Psychol. Sci. Soc. Sci..

[58] Treuhaft S., Karpyn A. (1992). Urban Grocery Gap.

[59] Wolfe W.S., Olson C.M., Kendall A., Frongillo E.A. (1996). Understanding food insecurity in the elderly: A conceptual framework. J. Nutr. Edu..

[60] Drywien M., Kuć A. (2019). Specyfika zachowań żywieniowych osób starszych pochodzących ze środowiska wiejskiego. Kosmos.

[61] Wądołowska L. (2010). Żywieniowe Podłoże Zagrożeń Zdrowia w Polsce.

[62] Weiseman M., Cannon G., Butrum R., Martin G., Higginbotham S., Heggie S., Jones C., Fletcher M. (2007). Food, Nutrition, Physical Activity and the Prevention of Cancer: A Global Perspective.

[63] Hung H.C., Joshipura K.J., Jiang R., Hu F.B., Hunter D., Smith-Warner S.A., Golditz G.A., Rosner B., Spiegelman D., Willet W.C. (2004). Fruit and vegetables intake and risk of major chronic disease. J. Natl. Cancer Inst..

[64] Lichtenstein A.H., Appel L.J., Brands M., Carnethon M., Danie L.S., Franch H.A., Franklin B., Kris-Etherton P., Harris W.S., Howard B. (2006). Diet and lifestyle Recommendations Revision 2006. A Scientific Statement from the American Heart Association Nutrition Committee. Cirulation.

[65] Stanner S., Thompson R., Buttris J.L. (2009). Healthy Aging: The Role of Nutrition and Life Style.

[66] Morland K., Wing S., Roux A. (2002). The contextual effect of the local food environment on residents’ diets: The atherosclerosis risk in communities study. Am. J. Public Health.

[67] CBOS Jakiej Pomocy Potrzebują Osoby Starsze i Kto Im Jej Udziela?. https://www.cbos.pl/SPISKOM.POL/2019/K_116_19.PDF.

[68] (2017). Usługi Opiekuńcze Świadczone Osobom Starszym w Miejscu Zamieszkania. https://www.nik.gov.pl/plik/id.17440.vp.20012.pdf.

[69] Fonseca-Centeno Z.Y., Álverez-Uribe M.C., Estrada-Restrepo A. (2010). Caracterización de los hogeres colombianos en inseguridad alimentaria según calided de vida. Revista de Salud Pública.

[70] (2019). Beneficjenci Środowiskowej Pomocy Społeczne w 2018 Roku. https://stat.gov.pl/obszary-tematyczne/warunki-zycia/ubostwo-pomoc-spoleczna/beneficjenci-srodowiskowej-pomocy-spolecznej-w-2018-roku,15,6.html.

[71] (2019). Rynek Wewnętrzy w 2018 Roku. https://stat.gov.pl/obszary-tematyczne/ceny-handel/handel/rynek-wewnetrzny-w-2018-roku,7,25.html.

[72] (2020). Sytuacja Gospodarstw Domowych w 2019 r. w Świetle Badania Budżetu Gospodarstw Domowych. https://stat.gov.pl/obszary-tematyczne/warunki-zycia/dochody-wydatki-i-warunki-zycia-ludnosci/sytuacja-gospodarstw-domowych-w-2019-r-w-swietle-badania-budzetow-gospodarstw-domowych,3,19.html.

[73] Diagnoza Społeczna 2015. Warunki i Jakość Życia Polaków. Raport. Warszawa 2016. http://www.diagnoza.com/pliki/raporty/Diagnoza_raport_2015.pdf.

[74] (2019). Regionalne Zróżnicowanie Jakości Życia w 2018 r. Wyniki Badania Spójności Społecznej 2018. https://stat.gov.pl/obszary-tematyczne/warunki-zycia/dochody-wydatki-i-warunki-zycia-ludnosci/regionalne-zroznicowanie-jakosci-zycia-w-polsce-w-2018-roku-wyniki-badania-spojnosci-spolecznej-2018,31,1.html.

[75] Majecka A., Nowak P. (2019). Uwarunkowania jakości życia w polskich województwach. Nierówności Społeczne a Wzrost Gospod..

[76] Quandt S.A., Arcury T.A., McDonald J., Bell R.A., Vitolins M.Z. (2001). Meaning and management of food security among rural elders. J. Appl. Gerontol..

[77] Ishiguro K. (2014). Food Access Among Elderly Japanese People. Asian Soc. Work Policy Rev..

[78] Nowakowski M., Kowaleski J.T., Szukalski P. (2004). Pojęcie wsparcia społecznego i problemy jego pomiaru ze szczególnym uwzględnieniem populacji seniorów. Nasze Starzejące się Społeczeństwo. Nadzieje i Zagrożenia.

[79] Trafiałek E. (2014). Rodzinna Jako Obszar Aktywności i Źródło Wsparcia w Aktywnym Starzeniu się. http://dspace.uni.lodz.pl/xmlui/handle/11089/4988.

[80] Błaszczyk-Bębenek E., Kostrz A., Szlegel-Zawadzka M. (2016). Ocena zdolności do samodzielnego funkcjonowania w życiu codziennym osób starszych z uwzględnieniem zachowań żywieniowych. Geriatria.

[81] Schlegel-Zawadzka M., Klich A., Kubik. B., Kołpa M. (2011). Ocena zdolności ludzi starszych do samoobsługi i samoopieki z uwzględnieniem zachowań żywieniowych. Pielęgniarstwo XXI Wieku.

[82] Fernandes S.G., Rodrigues A.M., Nunes C., Santos O., Gregório M.J., de Sousa R.D., Dias S., Canhão H. (2018). Food Insecurity in Older Adults: Results from the Epidemiology of Chronic Diseases Cohort Study 3. Front. Med..

[83] Bales C.W., Ritchie C.S. (2018). Handbook of Clinical Nutrition and Aging.

[84] Vilar-Compte M., Martínez-Martínez O., Orta-Alemán D., Perez-Escamilla R. (2016). Functional limitations, depression, and cash assistance are associated with food insecurity among older urban adults in Mexico City. J. Health Care Poor Underserved.

[85] Vilar-Compte M., Gaitán-Rossi P., Pérez-Escamilla R. (2017). Food insecurity measurement among older adults: Implications for policy and food security governance. Glob. Food Secur..

[86] Richard L.L., Gauvin C.G., Laforest S. (2009). Staying connected: Neighbourhood correlates of social participation among older adults living in an urban environment in Montreal, Quebec. Health Promot. Int..

[87] Moschis G. (2003). Marketing to older adults: An updated overview of present knowledge and practice. J. Consum. Market..

[88] Vaportzis E., Giatsi Clausen M., Gow A.J. (2017). Older Adults Perceptions of Technology and Barriers to Interacting with Tablet Computers: A Focus Group Study. Front. Psychol..

[89] Murrell A., Jones R. (2020). Measuring Food Insecurity Using the Food Abundance Index: Implications for Economic, Health and Social Well-Being. Int. J. Environ. Res. Public Health.

